# Growth in Hyper-Concentrated Sweet Whey Triggers Multi Stress Tolerance and Spray Drying Survival in *Lactobacillus casei* BL23: From the Molecular Basis to New Perspectives for Sustainable Probiotic Production

**DOI:** 10.3389/fmicb.2018.02548

**Published:** 2018-10-22

**Authors:** Song Huang, Floriane Gaucher, Chantal Cauty, Julien Jardin, Yves Le Loir, Romain Jeantet, Xiao Dong Chen, Gwénaël Jan

**Affiliations:** ^1^Suzhou Key Laboratory of Green Chemical Engineering, School of Chemical and Environmental Engineering, College of Chemistry, Chemical Engineering and Material Science, Soochow University, Jiangsu, China; ^2^UMR1253 STLO, Agrocampus Ouest, INRA, Rennes, France; ^3^Bioprox, Levallois-Perret, France

**Keywords:** osmoregulation, probiotics, lactic acid bacteria, stress response, physiology, label-free proteomics, spray-drying

## Abstract

*Lactobacillus casei* BL23 has a recognized probiotic potential, which includes immune modulation, protection toward induced colitis, toward induced colon cancer and toward dissemination of pathogens. In *L. casei*, as well as in other probiotics, both probiotic and technological abilities are highly dependent (1) on the substrate used to grow bacteria and (2) on the process used to dry and store this biomass. Production and storage of probiotics, at a reasonable financial and environmental cost, becomes a crucial challenge. Food-grade media must be used, and minimal process is preferred. In this context, we have developed a “2-in-1” medium used both to grow and to dry *L. casei* BL23, considered a fragile probiotic strain. This medium consists in hyper-concentrated sweet whey (HCSW). *L. casei* BL23 grows in HCSW up to 30% dry matter, which is 6 times-concentrated sweet whey. Compared to isotonic sweet whey (5% dry matter), these growth conditions enhanced tolerance of *L. casei* BL23 toward heat, acid and bile salts stress. HCSW also triggered intracellular accumulation of polyphosphate, of glycogen and of trehalose. A gel-free global proteomic differential analysis further evidenced overexpression of proteins involved in pathways known to participate in stress adaptation, including environmental signal transduction, oxidative and metal defense, DNA repair, protein turnover and repair, carbohydrate, phosphate and amino acid metabolism, and in osmoadaptation. Accordingly, HCSW cultures of *L. casei* BL23 exhibited enhanced survival upon spray drying, a process known to drastically affect bacterial viability. This work opens new perspectives for sustainable production of dried probiotic lactobacilli, using food industry by-products and lowering energy costs.

## Introduction

*Lactobacillus* is a major genus of the lactic acid bacteria (LAB), a GRAS (Generally Recognized as Safe) and economically important bacterial group used in foods, beverages, or dietary supplements. *Lactobacillus casei* is a facultative heterofermentative species usually used as a starter culture for milk fermentation and for the maturation of cheeses in the food industry (Hammes and Hertel, 2006). Selected certain strains of *Lactobacillus casei* can be used as probiotics (Hill et al., 2018). Promising data suggest a beneficial impact on several disorders in humans and in animals (Klaenhammer et al., 2012; Aktas et al., 2016).

*Lactobacillus casei* BL23, as an example, is a well-characterized probiotic. Its immunomodulatory properties were demonstrated *in vitro* (Bäuerl et al., 2011) and *in vivo* (Rochat et al., 2007; Lenoir et al., 2016; Cortes-Perez et al., 2017; Maiga et al., 2017; Qin et al., 2018). Its surface-exposed components were proposed to take part in interactions with the intestinal mucosa (Muñoz-Provencio et al., 2011; Qin et al., 2017), in inhibition of *Staphylococcus aureus* internalization (Bouchard et al., 2013; Souza et al., 2017), and in the metabolism of human milk oligosaccharides, which plays a key role in establishing and maintaining the infant gut microbiota (Bidart et al., 2015). Excreted microvesicles also carry key protein probiotic effectors of *L. casei* BL23 (Rubio et al., 2017) Consumption of *L. casei* BL23 modulates the microbiota in favor of *Lactobacillaceae*, *Porphyromonadaceae*, and *Comamonadaceae* (Yin et al., 2014).

To deliver probiotic benefits to the hosts, a stable probiotic product with a high bacterial viability is required (Tripathi and Giri, 2014). *L. casei* may be consumed under the form of fermented products, such as fermented olives, cheese or fermented milks. In this case, the food matrix plays a critical role in maintaining the viability of probiotics (Silva et al., 2015) and in protecting them from digestive assaults (Gagnaire et al., 2015; Rabah et al., 2018). As an alternative, *L. casei* may be consumed within probiotic food supplements. In this case, the galenic preparation should be designed to optimize viability, stability and stress tolerance of the probiotic (Broeckx et al., 2016). In this context, dried probiotic powders are favored by industries as active components for storage, transport and formulation (Huang et al., 2017). Probiotic powders are mainly produced by freeze-drying because of the low processing temperature and a maximum bacterial viability. Freeze-drying is, however, a discontinuous process, with growing, harvesting, resuspension, freezing and vacuum drying steps in a batch mode. Moreover, water removal implies to overcome several latent energy state changes (freezing, sublimation), thus leading to a high energy operating cost. By contrast, the spray-drying process, which is tougher toward bacteria, given the thermal and oxidative conditions, is a continuous process with a high productivity and energy operating cost twice to three times lesser than that of freeze-drying. It thus constitutes an alternative method to produce probiotic powders with higher productivity and energy efficiency (Huang et al., 2017). Whenever using freeze or spray drying, maintaining the viability of probiotics in a dry state remains a key challenge. Indeed, even if no strict dose-response effect is established by clinical studies (Ouwehand, 2017), probiotics human doses above 10 billion CFU per day are generally associated with a more significant study outcome (Kligler and Cohrssen, 2008). Digestive. stress tolerance of spray-dried probiotics remains a key issue. In this respect, growth and drying conditions can be driven in order to optimize probiotic efficacy (Broeckx et al., 2016; Huang et al., 2017).

*Lactobacillus casei* does not belong to the thermophilic lactic acid bacteria. It has a low constitutive tolerance toward oxidative stress, but which can be enhanced by habituation (Zotta et al., 2014) or by genetic engineering (Rochat et al., 2006). Several studies sought ways to enhance tolerance toward heat (Yang et al., 2017), acid (Alcántara et al., 2014) and bile salts (Xiong et al., 2017) stress in *L. casei*. Incubation of *L. casei* BL23 in milk prior to consumption promotes persistence in the mammalian digestive tract, further evidencing a profound influence of the growth medium on probiotic abilities (Lee et al., 2015a).

In accordance with above-cited studies, we have recently taken advantage of a dairy medium to produce probiotics with enhanced survival during technological and digestive stresses. We first studied the probiotic *Propionibacterium freudenreichii*. This cheese bacterium grows in Emmental cheese(a Swiss-type cheese) (Thierry et al., 1998), in the cheese aqueous extracts (Gagnaire et al., 2015) and in cheese whey, a side-product of the cheese industry. Interestingly, growth occurs not only in isotonic whey, but also in hyperconcentrated whey (Huang et al., 2016b). Such growth conditions induce accumulation of energy storage compounds, overexpression of stress adaptation proteins and multi-stress tolerance in propionibacteria. This led to the development of a novel process for spray drying of bacteria, using concentrated sweet whey, a by-product of Emmental cheese manufacture, as a “2-in-1” bacterial culture medium and spray drying matrix (Huang et al., 2017). It hence avoids the intermediate operation steps between fermentation and spray drying, resulting in a continuous production pattern and higher energy efficiency. Due to the high dry matter of concentrated sweet whey (30% w/w), this process allows spray-drying in relatively mild conditions with 130 – 140°C inlet temperature and 50 – 60°C outlet temperature, resulting in 100% *P. freudenreichii* survival rates at both laboratory scale and semi-industrial scale (Huang et al., 2017). We have previously investigated the stress adaptation of *P. freudenreichii* during growth in concentrated sweet whey. The osmoregulation of *P. freudenreichii* in concentrated sweet whey triggers the multistress response of bacteria, thus leading to the cellular tolerance against the extreme industrial spray-drying conditions. In a preliminary investigation, we have shown that *L. casei* BL23 could also be cultivated and dried using the same medium (Huang et al., 2017). However, the molecular mechanisms leading to multi-stress tolerance upon growth in hyperconcentrated whey remained to be investigated in *L. casei* BL23.

As shown in our previous work, *L. casei* is a relatively fragile species, compared to *P. freudenreichii* (Huang et al., 2017). Spray drying of such a fragile but widely commercialized species thus constitutes a challenging goal for both scientific and industrial stakeholders. Therefore, in this current work, the osmoadaptation, and the resulting multitolerance, were investigated in *L. casei* BL23 during growth in the concentrated sweet whey. A spray drying process in industrial condition was also applied to further explore the feasibility of producing osmoadapted *L. casei* via the novel spray drying process. We furthermore compared different concentrations of whey to define optimal conditions.

## Materials and Methods

### Strains and Pre-culture

*Lactobacillus casei* BL23 was provided by UMR1219 MICALIS, (INRA-AgroParisTech, Jouy-en-Josas, France), stored and maintained by the CIRM-BIA Biological Resource Center (Centre International de Ressources Microbiennes-Bactéries d’Intérêt Alimentaire, INRA, Rennes, France). The pre-culture of *L. casei* was prepared routinely by inoculation (1% inoculum size) of MRS broth and static cultivation at 37°C for 16 h.

### Bacterial Growth in Sweet Whey

Growth of *L. casei* in sweet whey medium was performed as described (Huang et al., 2016a). Briefly, sweet whey media were prepared by rehydration of sweet whey powder (Lactalis ingredients, Mayenne, France) in deionized water to obtain the media with final total solids content (TS, w/w) of 5, 10, 20, 30, and 40%, respectively. These culture media were then autoclaved at 100°C for 30 min before inoculation (1% inoculum size) with *L. casei* from the MRS pre-culture. The inoculated culture media were incubated statically at 37°C for 48 h. The growth curve of *L. casei* in sweet whey was monitored by CFU counting on plates of MRS medium solidified with 10 g/liter agar.

### Stress Challenges

*Lactobacillus casei* was cultivated 48 h in 5, 20, 30, and 40% total solid content sweet whey, as described above. These different cultures were stressed as follows. Heat challenge was applied to *L. casei* by placing 1.2 mL (in 2 mL Eppendorf tube) of the *L. casei* sweet whey culture in a water bath at 60°C for 10 min, as described previously (Leverrier et al., 2004). Bile salts challenge was performed by adding 1 g/liter of bile salts (an equimolar mixture of cholate and deoxycholate; Sigma Chemical, St. Louis, MO, United States) in the culture at 37°C for 60 min as described (Leverrier et al., 2003). Acid challenge was performed by re-suspending the *L. casei* cell pellets in MRS broth adjusted to pH 2.0 using HCl, at 37°C for 60 min as described (Jan et al., 2000). The viability of *L. casei* was determined by CFU counting on MRS-agar. As a control, cultures were left untreated at 37°C for the same time in order to determine the population corresponding to 100% survival. Percent survival was calculated by comparing stressed and unstressed cultures at the end of the same time.

### Microscopy

#### Light Microscopy

Neisser staining was carried out to observe the polyphosphate granule accumulated in *L. casei* cells in 5% and 30% sweet whey culture according to the procedure by (Alcántara et al., 2014). Bacterial cultures were heat fixed on microscope slides and then covered for 1 min with a freshly prepared mixture of 1 volume of Neisser’s methylene blue solution (Fluka Analytical, France) and 2 volumes of 0.33% crystal violet solution in 10% ethanol. Excess dye was absorbed using blotting paper, and the slides were covered for 1 min with a 0.3% solution of chrysoidin G solution (Sigma–Aldrich, Saint Quentin Fallavier, France) prior to extensive rinsing with deionized water. The slides were observed on an Olympus BX51 light microscope at a × 1,000 magnification.

#### Epifluorescence Microscopy

Polyphosphate granules were also visualized by DAPI (4′,6-diamidino-2-phenylindole) staining as previously described by Mukherjee et al. (2015). Briefly, bacteria were washed in McIlvaine’s buffer, fixed in 4% formaldehyde, permeabilized in 0.3% Triton X-100, and then stained by 20 μg/mL DAPI in the same buffer. The stained cultures were observed on an Olympus BX51 epifluorescence microscope equipped with aU-MWU2 fluorescence filter cube (excitation filter, 330–385 nm; emission filter, 480–800 nm) and an Olympus plan 100×/1.25 oil objective.

#### Transmission Electron Microscopy

The morphology of *L. casei* in 5 and 30% sweet whey culture was visualized by transmission electron microscopy. Briefly, osmolarity of the 5 and 30% sweet whey culture was firstly quantified by a freezing-point osmometer (Osmomat 030-D, Gonotec, Berlin, Germany). The *L casei* cell pellets were collected from 5 or 30% sweet whey culture by centrifugation at 8000 *g* for 10 min. These cell pellets were then washed and re-suspended in a saline buffer with the same osmolarity as their previous culture prior to fixation with 2.5% glutaraldehyde for 3 h. The bacterial pellets were embedded in agar prior to being cut into 1-mm pieces and fixed with 1% sodium tetroxide for 1 h. The agar pieces were rinsed with cacodylate buffer and dehydrated in ethanol prior to inclusion in Epon-Araldite-DMP30 resin (polymerized at 60°C for 48 h). Thin sections (90 nm) were cut (Leica ultra-microtome Ultracut E), collected on copper grids, and then stained with uranyl acetate. Samples were observed with a Jeol 1400 electron microscope (Jeol Co. Ltd., Tokyo, Japan), and images were digitally captured with a Gatan Orius camera (Digital Micrograph Software). The bacterial cell diameter and cell wall thickness were then quantified using ImageJ^1^. Cells with clear edge were selected for the measurement. A total of 20 or more cells from each group were measured.

### Measurement of Intracellular Glycogen and Trehalose

The intracellular trehalose and glycogen were quantified for *L. casei* in the 5 and 30% sweet whey culture as described previously with slight modification (Huang et al., 2016b). Briefly, *L. casei* cell pellets were washed twice by PBS and then divided into two parts, one for quantification of *L. casei* cell number by CFU counting on MRS agar plates, and the other one for quantification of trehalose and glycogen. The latter sample was re-suspended in acetate buffer (40 mM, pH 5.2) after washing twice by PBS. The cell suspension was then heat inactivated for 5 min at 95 °C, and disrupted using zirconium beads in a homogenizer (Bertin Technologies, Toulouse France) prior to centrifugation of cellular debris. The resulting extract was divided into two parts, further digested using amyloglucosidase (from *Aspergillus niger* [no. 10115; Sigma–Aldrich]) or trehalase (from porcine kidney [no. T8778; Sigma-Aldrich]) for the hydrolysis of glycogen and trehalose, overnight at 57°C and 37°C, respectively. Samples were quickly frozen, and the resulting glucose was quantified using a glucose hexokinase assay kit (Sigma-Aldrich). The results of glycogen and trehalose concentrations were expressed and normalized as the concentration of generated glucose per 10^10^ cells (CFU).

### Label-Free Proteomics

#### Whole-Cell Protein Extraction and Tryptic Digestion of Proteins

The whole-cell proteins were extracted as described before (Huang et al., 2016b). *L. casei* in 5 and 30% sweet whey cultures were harvested by centrifugation. In order to maximally exclude the influences of sweet whey components, the cell pellets were washed twice by 0.1 M Tris-citrate buffer (pH 8, containing 2% sodium citrate), twice by 0.1 M Tris-EDTA buffer (pH 8, containing 0.002 M EDTA), and by PBS buffer. Cell pellets were then re-suspended in SDS lysis buffer and frozen for 2 h prior to sonication and cell lysis using zirconium beads in the homogenizer. The resulting SDS extracts were recovered by centrifugation (21,000 ×*g*; 20°C; 20 min) and then cleaned and quantified using the two-dimensional (2-D) clean-up kit (GE Healthcare Bio-Sciences AB, Uppsala, Sweden) and the 2-D quant kit (GE Healthcare Bio-Sciences AB, Uppsala, Sweden), respectively. Tryptic digestion was performed on 100 μg proteins from each sample overnight at 37°C and stopped with spectrophotometric-grade trifluoroacetic acid (TFA) (Sigma–Aldrich) as described previously (Leverrier et al., 2004). The supernatants containing peptides were then vacuum dried in Speed-Vac concentrator and stored at -20°C until mass spectrometry analysis.

#### Nano-LC-MS/MS

Experiments were performed using a nano RSLC Dionex U3000 system fitted to a Q-Exactive mass spectrometer (Thermo Scientific, San Jose, CA, United States) equipped with a nano-electrospray ion source. A preliminary sample concentration step was performed on a C18 pepMap100 reverse-phase column [C18 column, 300-μm inner diameter (i.d.) by 5 mm length, 5 μm particle size, 100 Å pore size; Dionex, Amsterdam, Netherlands]. Peptide separation was performed on a reversed-phase column (PepMap RSLC C18, 75 μm i.d. by 250 mm length, 3 μm particle size, 100 Å pore size; Dionex) with a column temperature of 35°C, using solvent A [2% (v/v) acetonitrile, 0.08% (v/v) formic acid and 0.01% (v/v) TFA in deionized water] and solvent B [95% (v/v) acetonitrile, 0.08% (v/v) formic acid and 0.01% (v/v) TFA in deionized water]. Peptides were separated using a gradient of 5–35% solvent B over 80 min followed by 35 to 85% solvent B over 5 min at a flow rate of 0.3 μL/min. Eluted peptides were directly electro sprayed into the mass spectrometer operating in positive ion mode with a voltage of 2 kV. Spectra were recorded in full MS mode and selected in a mass range 250–2000 m/z for MS spectra with a resolution of 70,000 at m/z 200. For each scan, the ten most intense ions were selected for fragmentation. MS/MS spectra were recorded with a resolution of 17,500 at m/z 200 and the parent ion was subsequently excluded from MS/MS fragmentation for 20 s. The instrument was externally calibrated according to the supplier’s instructions.

#### Protein Identification

Peptides were identified from the MS/MS spectra using X!Tandem pipeline software (Langella et al., 2017). The search was performed against a database composed of (i) an in-house database composed of major milk and egg proteins derived from www.uniprot.org (207 proteins in total) and (ii) a portion of the UniProtKB database corresponding to *L. casei* BL23^2^. Database search parameters were specified as follow: trypsin cleavage was used and the peptide mass tolerance was set to 10 ppm for MS and 0.05 Da for MS/MS. Oxidation of methionine and phosphorylation on threonine, serine and tryptophan were selected as a variable modification. For each peptide identified, a minimum score corresponding to an *e*-value below 0.05 was considered as a prerequisite for peptide validation.

#### Protein Quantification

Every peptide identified by tandem mass spectrometry was quantified using the free MassChroQ software (Valot et al., 2011) before data treatment and statistical analysis within the R software (R 3.2.2, Project for statistical computing). A specific R package called ‘MassChroqR’ was used to automatically filter dubious peptides for which standard deviation of retention time was superior to 40 s and to regroup peptide quantification data into proteins. Two different and complementary methods of analysis were used, based on peak counting or XIC (eXtracted Ion Current). For peak counting analysis, analysis of variance was performed on proteins with a minimum peak ratio of 1.5 between both culture conditions. Proteins with an adjusted *p*-value < 0.05 were considered significantly different.

For XIC based quantification, normalization was performed to take into account possible global quantitative variations between LC-MS runs. Peptides shared between different proteins were automatically excluded from the data set as well as peptides present in less than 80% of samples. Missing data were then imputed from a linear regression based on other peptide intensities for the same protein (Blein-Nicolas et al., 2015). Analysis of variance was used to determine proteins with significantly different abundance between our two culture conditions.

### Spray-Drying Application

Spray drying was performed on a laboratory-scale spray dryer (Mobile Minor^TM^, GEA Niro, Denmark). *L. casei* was cultivated in 5, 20, and 30% total solid content sweet whey, as described above. These different cultures (1∼2 l) of *L. casei* were agitated for 10 min prior to delivery to the dryer by a peristaltic pump (520S, Watson-Marlow, France). A two-fluid nozzle with a diameter of 0.8 mm was used for atomization. The inlet air temperature was fixed at 185°C. The temperature and relative humidity of outlet air were controlled at 75 ± 2°C and 10 ± 1%, respectively, by adjusting the feed rate. The bacterial viabilities before and after spray drying were estimated by enumeration on MRS agar plates. The bacterial population and percentage of survival in powders were obtained as described previously (Huang et al., 2016a).

### Statistical Analysis

All the results are presented as mean value with standard deviation. The data were from triplicate samples. The analysis of variance (ANOVA) followed by Tukey’s test was performed using R software with the ‘Rcmdr’ package (R Development Core Team). Differences between mean values were considered significant when *p* < 0.05.

## Results

### Bacterial Growth in Sweet Whey Media

**FIGURE 1:**
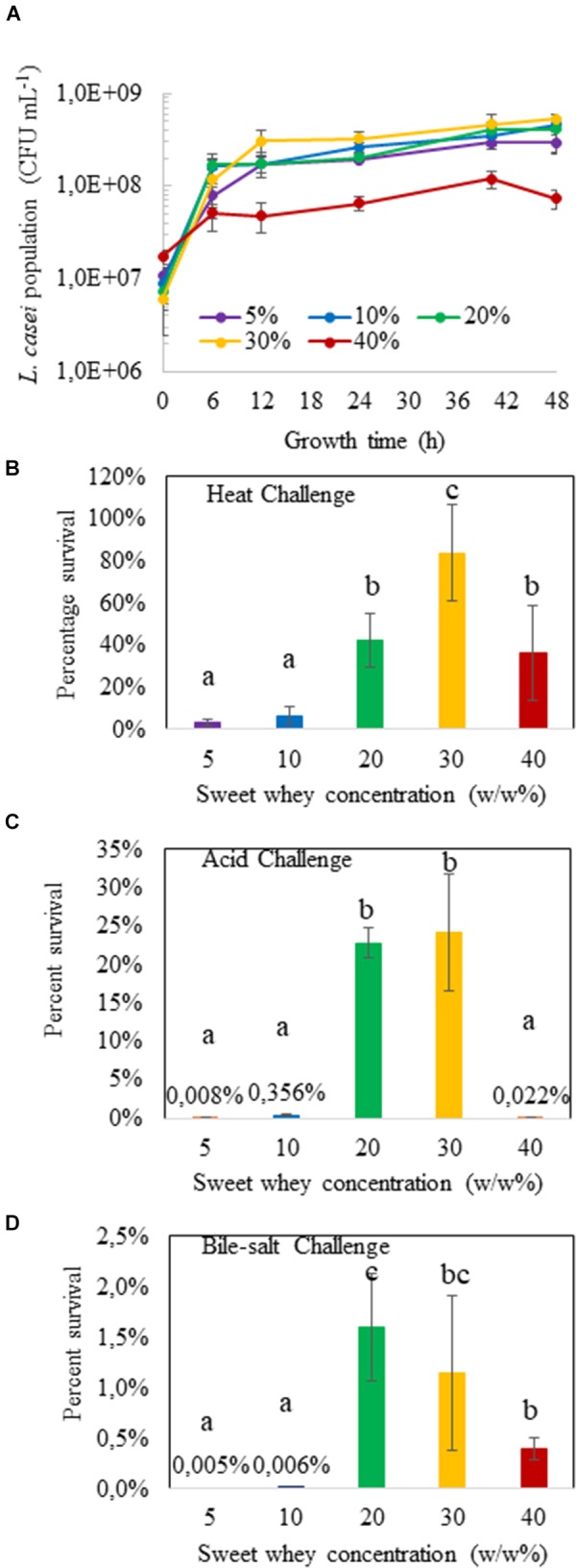
Hyperconcentrated sweet whey culture confers stress tolerance to *L. casei* BL23. Lactobacilli were cultivated 48 h in sweet whey at different concentrations until stationary phase and their population determined by CFU counting **(A)**. They were then subjected to heat (**B**, 60 °C for 10 min), acid (**C**, pH 2.0 for 1 h) or bile salts (**D**, 1 g/L for 1 h) challenge as described in materials and methods. Viable lactobacilli were enumerated by plate counting in treated and control cultures. Results are expressed as percent survival. Error bars represent the standard deviation for triplicate experiments. Different letters above the columns mean significant difference (*p* < 0.05).

The growth curves of *L. casei* BL23 in sweet whey media are shown on Figure 1A. Sweet whey in the concentration ranging from 5 to 30% dry matter sustained the growth of *L. casei* inoculated at 10^7^ CFU/mL with a stationary phase approximately starting at 12 h and a final population close to 2 × 10^8^ CFU/mL, for 5, 10, 20, and 30% sweet whey. However, final population was slightly lower in 40% sweet whey, close to 10^8^ CFU/mL. *L. casei* in 30% sweet whey yielded the highest final bacterial population, among all the cultures. The resulting population was 3 × 10^8^ CFU/mL, twice that of 5% sweet whey cultures (*p* = 0.063, 0.071 and 0.008 at 12, 24, and 48 h respectively).

### Bacterial Stress Physiology

The different *L. casei* BL23 sweet whey cultures, 48 h of incubation, were challenged with heat, acid or bile salts stresses, respectively. Culture in hyperconcentration of sweet whey clearly induced multitolerance. At sweet whey concentrations of 5 and 10%, whatever the challenge, percent survival was close to the lower limit of detection (Figures 1B–D). However, *L. casei* cultured in 20 and 30% sweet whey displayed an enhanced stress tolerance, in comparison to the isotonic 5% sweet whey culture. For instance, the survival rates of *L. casei* in 30% sweet whey culture under heat, acid and bile-salt stress were approximately 80, 25, and 1%, respectively, while the survival rates were only 3, 0.008, and 0.005% in the 5% sweet whey culture. Survival rates of *L. casei* cultured in 40% sweet whey were lower than in 30% sweet whey culture. This result indicates the multi-stress tolerant phenotype of *L. casei* in the 20 and 30% sweet whey cultures.

### Accumulation of Intracellular Polyphosphate and Sugar

**FIGURE 2:**
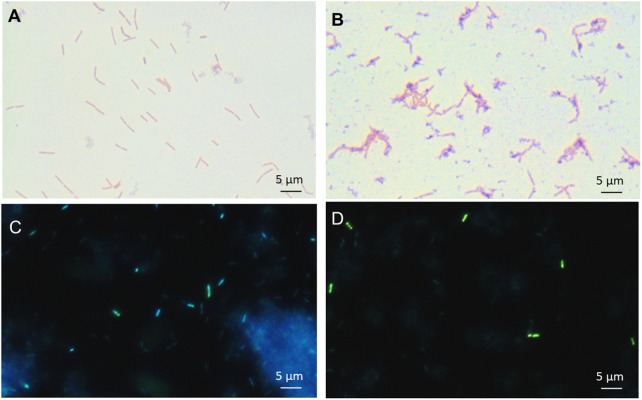
Hyperconcentrated sweet whey triggers intracellular accumulation of polyphosphate granules in *L. casei* BL23. Lactobacilli were cultivated 48 h in isotonic 5% **(A,C)** or hyperconcentrated 30% **(B,D)** sweet whey. Polyphosphate metachromatic granules were evidenced by Neisser staining of fixed culture smears prior to microscopy observation at x1000 magnification **(A,B)**. Polyphosphate was visualized by DAPI staining prior to epifluorescence microscopy **(C,D)** observation. Green fluorescence indicates cytosolic poly P and blue fluorescence DNA. The scale bar indicates the length corresponding to 5 μm.

Accumulation of intracellular inorganic polyphosphate was sought using both Neisser staining and DAPI staining for the *L. casei* cells grown in isotonic (5%) and hyperconcentrated (30%) sweet whey. Polyphosphate accumulation under the form of dark-stained granules was observed, as reported for various *Lactobacillus* species using Neisser staining (Alcántara et al., 2014). These dark granules were abundantly observed in the 30% sweet whey *L. casei* BL23 cultures, yet rarely in 5% sweet whey cultures (Figures 2A,B). This result was confirmed using DAPI staining. The green fluorescence of bacteria (Figures 2C,D) indicated the abundance of polyphosphate accumulated inside *L. casei* cells (Mukherjee et al., 2015).

In addition to the accumulation of polyphosphate, the amounts of intracellular trehalose and glycogen were also quantified for the 5 and 30% *L. casei* sweet whey cultures. Glycogen significantly increased (*p* < 0.05) from 11.12 (SD 3.00) to 42.92 (SD6.24) glucose eq. [μg/1010 cells] when increasing the sweet whey dry matter from 5 to 30%. Accordingly, trehalose increased significantly (*p* < 0.05) from 54.58 (SD 7.63) to 126.14 (SD 27.19) glucose eq. [μg/1010 cells]. Cells thus accumulated around 4 times more intracellular glycogen and twice more intracellular trehalose, in comparison to 5% sweet whey cultures.

### Characterization of Bacterial Cell Morphology

**FIGURE 3:**
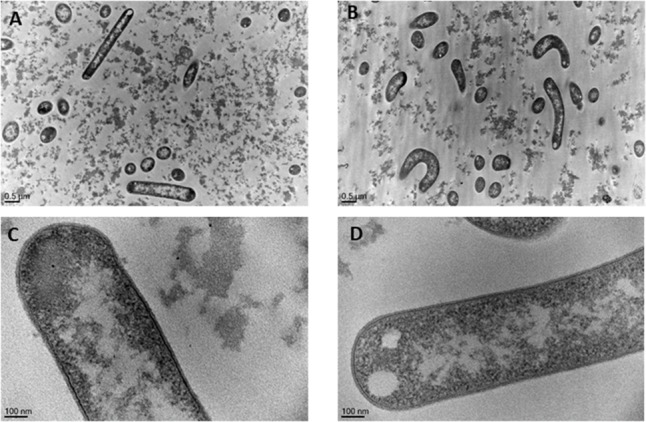
Hyperconcentrated sweet whey triggers morphological rearrangements in *L. casei* BL23. Lactobacilli were cultivated 48 h in isotonic 5% **(A,C)** or hyperconcentrated 30% **(B,D)** sweet whey. They were then fixed, stained, and ultrathin sections were observed using transmission electron microscopy. The bacterial cell diameter and cell wall thickness were then quantified. Cells with clear edge were selected for the measurement.

Morphology of *L. casei* grown in 5% sweet whey and 30% sweet whey was compared using transmission electron microscopy (Figure 3). *L. casei* cells in 5% sweet whey (Figures 3A,C) displayed a typical rod shape of lactobacilli. However, the cells in 30% sweet whey had a distorted morphology (Figures 3B,D) with a visible curvature. There was no significant difference between the cell diameters of *L. casei* grown in 5 and 30% sweet whey. However, the *L. casei* BL23 cells grown in 30% sweet whey had a significantly thinner cell wall. Indeed, the thickness varied from 19.32 nm (SD 4.60) to 10.90 nm (SD 1.40), when increasing the sweet whey dry matter from 5 to 30%.

### Upregulation of Proteins in the 30% Sweet Whey Culture

The proteomic differential analysis focused on proteins which were overexpressed in 30% sweet whey, with a minimum ratio of 1.5. A total of 54 proteins were significantly up-regulated (>1.5 fold, *P* < 0.05) in the 30% *L. casei* sweet whey cultures, as compared to the 5% sweet whey cultures (Supplementary Figure 1). These proteins were categorized according to their biological process and molecular function in Uniprot database, or their metabolism pathways in KEGG classification (Table 1). Among these 54 proteins, 18 proteins (33.3%) were involved in the global stress response, including 3 proteins for DNA replication, recombination and repair, one transcription elongation factor, 3 proteins for environmental signal transduction, 4 proteins for oxidative and metal defense, and 7 proteins with chaperone properties.

**Table 1:** Proteins overexpressed after growth in hyperconcentrated 30% sweet whey, as compared with isotonic 5% sweet whey.

Uniprot accession	Description	Ratio 30/5	*p*-value^b^	Log (*e*-value)
**Transcription regulation**				
tr|B3WF14|B3WF14_LACCB	Transcription elongation factor GreA	3,03	3,2E-08	–48.4
**Environmental signal transduction**				
sp|B3WCP4|HPRK_LACCB	HPr kinase/phosphorylase	1,59	2,7E-03	–7.3
tr|B3W9M7|B3W9M7_LACCB	Similar to universal stress protein, UspA family	1,92	3,7E-04	–32.2
tr|B3WF83|B3WF83_LACCB	Beta-lactamase-like protein	2,43	3,4E-06	–9.0
**Oxidative and metal defense mechanisms**				
tr|B3WDN3|B3WDN3_LACCB	Conserved protein involved in Fe/S cluster assembly (GN = sufD)	1,53	3,5E-03	–11.2
tr|B3W8K6|B3W8K6_LACCB	Oxidoreductase (2,5-diketo-D-gluconic acid reductase)	1,66	7,5E-03	–71.8
tr|B3W8V4|B3W8V4_LACCB	Possible flavin reductase	2,22	1,9E-04	–11.4
tr|B3WEZ9|B3WEZ9_LACCB	Aluminum resistance protein	2,10	3,2E-05	–9.9
**Posttranslational modification, protein turnover, chaperones**				
sp|B3W9W8|CH10_LACCB	10 kDa chaperonin (GN = groS)	1,66	6,5E-04	–118.1
tr|B3WEQ8|B3WEQ8_LACCB	Protein GrpE	1,93	3,2E-06	–45.9
sp|B3WEQ7|DNAK_LACCB	Chaperone protein DnaK	2,29	2,4E-06	–281.7
**Carbohydrate metabolism**				
tr|B3WC28|B3WC28_LACCB	Malolactic enzyme	+∞^a^	0.2E-03	–8.5
tr|B3WAP8|B3WAP8_LACCB	L-lactate dehydrogenase	1,78	2,7E-03	–293.9
tr|B3WBW7|B3WBW7_LACCB	Tagatose-6-phosphate kinase	2,22	4,2E-02	–23.8
tr|B3WC62|B3WC62_LACCB	Glucose-6-phosphate 1-dehydrogenase	2,72	2,9E-05	–39.7
tr|B3W7I6|B3W7I6_LACCB	UDP-glucose 4-epimerase (Galactowaldenase) (UDP-galactose 4-epimerase)	2,88	1,7E-06	–80.1
tr|B3WBT7|B3WBT7_LACCB	Glucosamine-6-phosphate deaminase	3,91	8,9E-04	–96.1
tr|B3WBV8|B3WBV8_LACCB	Aldose 1-epimerase (Mutarotase), GN = galM	4,40	6,5E-09	–21.2
tr|B3WEK0|B3WEK0_LACCB	Putative 4-oxalocrotonate tautomerase (4-OT)	3,24	2,7E-07	–27.9
tr|B3WBW2|B3WBW2_LACCB	Phosphotransferase system sugar-specific EII component	5,91	5,1E-08	–66.2
**Amino acid metabolism**				
tr|B3WC99|B3WC99_LACCB	Xaa-His dipeptidase V (Carnosinase)	1,56	5,0E-03	–25.8
tr|B3WC34|B3WC34_LACCB	Peptidase M3B, oligoendopeptidase F	1,68	1,4E-02	–13.6
tr|B3WDM6|B3WDM6_LACCB	Glycine cleavage system H protein	1,80	1,2E-04	–21.4
tr|B3W7R6|B3W7R6_LACCB	Cysteine synthase	2,66	8,6E-04	–347.1
**Nucleic acid metabolism, DNA replication, recombination and repair**				
tr|B3W6R5|B3W6R5_LACCB	Single-stranded DNA-binding protein	1,62	7,7E-04	–36.6
tr|B3WEH2|B3WEH2_LACCB	Ribonucleoside-diphosphate reductase	1,97	2,4E-03	–16.4
tr|B3WEH3|B3WEH3_LACCB	Ribonucleoside-diphosphate reductase, beta chain	2,47	8,7E-07	–21.7
**Translation, ribosomal structure, protein synthesis**				
sp|B3WF43|RL20_LACCB	50S ribosomal protein L20	1,65	2,0E-06	–14.3
tr|B3WES1|B3WES1_LACCB	Proline–tRNA ligase	1,70	1,1E-02	–18.3
sp|B3WE38|EFTU_LACCB	Elongation factor Tu	1,73	8,5E-05	–311.3
tr|B3WEY7|B3WEY7_LACCB	Elongation factor P	2,47	1,8E-05	–20.3
sp|B3WES7|EFTS_LACCB	Elongation factor Ts	5,27	1,6E-02	–95.2
tr|B3WE79|B3WE79_LACCB	30S Ribosomal protein S1	1,98	7,3E-06	–135.9
sp|B3WD58|GATB_LACCB	Aspartyl/glutamyl-tRNA(Asn/Gln) amidotransferase subunit B	2,07	9,3E-05	–33.9
tr|B3WD56|B3WD56_LACCB	Aspartyl/glutamyl-tRNA(Asn/Gln) amidotransferase subunit C	2,30	2,1E-05	–18.9
sp|B3WES5|RRF_LACCB	Ribosome-recycling factor	2,75	1,7E-07	–31.6
**Phosphate-containing compound metabolic process**				
tr|B3WEB7|B3WEB7_LACCB	Inorganic pyrophosphatase	2,90	1,6E-02	–123.1
**Coenzyme/cofactor biosynthesis**				
tr|B3WEK1|B3WEK1_LACCB	Putative GTP cyclohydrolase 1 type 2	1,95	2,7E-04	–10.2
**Transporter**				
tr|B3WDN2|B3WDN2_LACCB	ABC transporter ATP-binding protein, GN = ysfB	1,89	4,2E-04	–11.6
tr|B3WDQ6|B3WDQ6_LACCB	Oligopeptide ABC transporter, substrate-binding lipoprotein (GN = oppA)	2,81	2,9E-04	–9.2
**Cell wall/membrane/envelope proteins**				
tr|B3WAU6|B3WAU6_LACCB	Putative integral membrane protein yqhA, GN = yqhA	2,73	1,8E-07	–35.4
tr|B3W7Q5|B3W7Q5_LACCB	Membrane alanine aminopeptidase (pepN)	2,07	8,5E-05	–28.9
**Function unknown**				
tr|B3WEQ2|B3WEQ2_LACCB	Putative uncharacterized protein	1,68	1,1E-03	–27.8
sp|B3W7G6|Y711_LACCB	UPF0145 protein	1,74	9,1E-03	–21.1
tr|B3WE90|B3WE90_LACCB	Putative uncharacterized protein, GN = LCABL_16100	1,81	9,5E-03	–14.4
tr|B3W7L7|B3W7L7_LACCB	Putative uncharacterized protein	1,90	6,5E-04	–14.4
tr|B3WCC5|B3WCC5_LACCB	Putative uncharacterized protein, GN = LCABL_09320	1,92	2,9E-05	–13.4
tr|B3W9N9|B3W9N9_LACCB	Putative uncharacterized protein yfhL	1,92	8,9E-04	–21.5
tr|B3W7L3|B3W7L3_LACCB	Putative uncharacterized protein	1,94	1,3E-04	–13.8
tr|B3W9U7|B3W9U7_LACCB	Putative uncharacterized protein, GN = LCABL_23990	2,18	6,8E-06	–4.4
tr|B3WCK6|B3WCK6_LACCB	Uncharacterized protein ywcC	2,24	3,3E-07	–49.2
tr|B3WA70|B3WA70_LACCB	Putative uncharacterized protein, GN = LCABL_25230	2,45	6,6E-06	–30.0
tr|B3W7M0|B3W7M0_LACCB	Putative uncharacterized protein, GN = LCABL_01870	3,38	1,3E-08	–67.6
tr|B3WEG9|B3WEG9_LACCB	Putative uncharacterized protein, GN = LCABL_16890	4,10	3,1E-07	–8.3


*^a^Ratio 30/5 is + ∞ when the protein is only expressed in bacteria grown in 30% sweet whey. ^*b*^Proteins with a p-value < 0,05, when comparing abundance in bacteria grown in 5% versus grown in 30% sweet whey, were considered as significantly different between 5% and 30% sweet whey cultures.*

Apart from the proteins relevant to global stress response, 9 proteins (16.7%) were involved in carbohydrate metabolisms, including a malolactic enzyme which is only presented in 30% sweet whey culture. Besides, except the 12 uncharacterized proteins, the other proteins are related to amino acid metabolism, translation and ribosomal structure, phosphate-containing compound metabolism, coenzyme/cofactor biosynthesis, transporter, and cell wall/membrane/envelope proteins.

### Application in Spray-Drying

**FIGURE 4:**
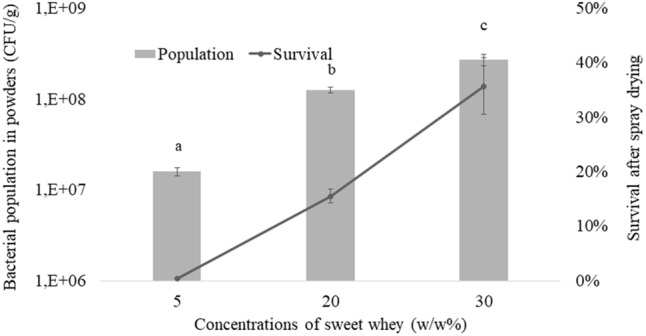
Hyperconcentrated sweet whey improves *L. casei* BL23 survival during spray drying. Lactobacilli were cultivated 48 hours in isotonic (5%) or hyperconcentrated (20 and 30%) sweet whey. These different cultures were then subjected to spray drying. Live cells were then enumerated in the resulting powders upon rehydratation, by CFU counting. Lactobacilli populations, in CFU per gram of powder, were calculated (left ax). Survival, when comparing live populations before and after spray drying, is reported (right ax).

Multi-tolerance being evidenced, as a result of *L. casei* BL23 growth in hyperconcentrated sweet whey, we then investigated its tolerance toward spray drying, as this technique is reputed as a severe challenge for bacteria. As shown on Figure 4, survival was close to 0 when *L. casei* BL23 5% sweet whey cultures were subjected to spray-drying. However, it was close to 20% and to 40% when the same bacterium was cultured in 20 and 30% dry matter sweet whey, respectively. This indicates a dose-dependent relation between medium concentration and survival rate during spray drying. Accordingly, when cultured in 30% sweet whey, the BL23 concentration was close to 10^9^ CFU per gram in the resulting powder, while it was close to 10^7^ CFU per gram with 5% sweet whey cultures.

## Discussion

*Lactobacillus casei* BL23 is probiotic bacterium which modulates cytokines secretion (Foligné et al., 2007), protects from chemically induced colitis (Rochat et al., 2007), colorectal cancer (Lenoir et al., 2016; Jacouton et al., 2017), or cancer chemotherapy-induced colitis (Cordeiro et al., 2018) in animal models, in accordance with its immunomodulatory ability (Lenoir et al., 2016; Cortes-Perez et al., 2017). Protection toward colitis depends on the dairy delivery matrix in which *L. casei* BL23 is consumed (Lee et al., 2015b). In addition, a dairy growth medium modulates this potential by enhancing persistence within the digestive tract, in accordance with an enhanced expression of key factors, such as surface proteins, involved in probiotic action (Lee et al., 2015a). Altogether, these data point to the critical role played by the nature of both the growth medium used for industrial production of *L. casei* BL23, and of the food delivery vehicle used for human consumption. In the present report, we thus investigated the potential of sweet whey, a dairy by-product of pressed cheese manufacture, to produce food-grade dried preparations comprising toughened live cells of *L. casei* BL23.

This last was shown here to grow on food-grade sweet whey. Industrial whey has already been used as a carrier to dry lactobacilli (Lavari et al., 2017) and other probiotics and food grade bacteria (Huang et al., 2017). However, we show here for the first time that growth of *L. casei* BL23 occurs in sweet whey in a wide range of concentrations, from 5% dry matter, which is the isotonic concentration, up to 40%, 8 times the isotonic concentration, leading to an osmotic pressure above 50 bar. This suggests efficient adaptation mechanisms leading to yet unrevealed stress tolerance in this bacterium, which is mesophilic and mainly encountered in the digestive tract of mammals. Interestingly, adaptation to the hyperosmotic sweet whey led to the acquisition of tolerance, by a cross-protection induction, toward key stresses encountered during technological and digestive processes (heat, acid, bile salts). These results also suggest that, since BL23 better survives to spray drying process when cultured on 30% sweet whey, this culture condition prepares the bacterium to the oxidative stress of the spray drying process.

Surprisingly, exposition to salt stress was previously reported to induce alterations in the cell wall of *L. casei* BL23, together with enhanced sensitivity to stressing agents such as mutanolysin, lysozyme, nisin and antibiotics (Piuri et al., 2005; Palomino et al., 2013). Cross protection, and the corresponding mechanisms, thus deserve investigation in osmoadapted *L. casei* BL23.

Osmoadaptation, i.e., the intracellular readjustments induced by hyperosmotic conditions, is most probably the main stimulus leading to multiple tolerance, as a result of *L. casei* BL23 growth in hyper concentrated sweet whey. Osmoadapatation has already been shown to induce cross protection toward various stresses in other bacteria (Papadimitriou et al., 2016). However, conflicting results are reported, as a function of the growth medium. As an example, salt adaptation induces tolerance toward bile salts and heat challenge in *Enterococcus faecalis* (Flahaut et al., 1996), but only in specific conditions. The availability of the osmoprotectant glycine betaine was further shown to drastically affect cross protection in the same bacterium (Pichereau et al., 1999). Not only osmotic stress, but also the nature of accumulated intracellular solutes, determine tolerance acquisition in bacteria (Papadimitriou et al., 2016). We thus investigated known intracellular solutes, generally accumulated under stress adaptation. Indeed, trehalose, glycogen and polyphosphate were accumulated here upon growth in hyperconcentrated sweet whey. Trehalose is a well-known intracellular compatible solute involved in osmoadaptation, but also stored as a reserve compound, mainly in vegetative resting cells, in many bacteria and in yeasts (Argüelles, 2000). In lactobacilli, little is known about its accumulation in response to hyperosmotic stress. However, when exogenously provided, it affords enhanced tolerance of lactobacilli toward heat, cold, dehydration, freeze-drying and spray-drying (Bravo-Ferrada et al., 2015; Zheng et al., 2016). Its accumulation within *L. casei* BL23 cells thus played a role in enhanced multi-tolerance upon growth in hyperconcentrated sweet whey. Glycogen was also accumulated in our conditions. Although it does not affect intracellular osmotic pressure, it plays a role in adaptation. The complete metabolic pathway for glycogen biosynthesis and accumulation, was found in lactobacilli selected species predominantly associated with mammalian and natural habitats, including *L. casei*. This biosynthesis plays a key role in tolerance toward bile and in probiotic fitness within the digestive tract (Goh and Klaenhammer, 2014). Polyphosphate was also accumulated within cells of *L. casei* BL 23, in the hyperconcentrated sweet whey, yet not in the isotonic one. Accumulation of polyphosphate was already reported in several *Lactobacillus* species, including *casei* (Alcántara et al., 2014). As reported by these authors, polyphosphate accumulates within the cytoplasm under the form of granules. This accumulation depends on the availability of elevated concentrations of phosphate in the medium and on the presence of polyphosphate kinase (*ppk*) gene. It is involved in stress resistance and disruption of *ppk* leads to reduced survival. Accordingly, polyphosphate storage plays a key role in the thermotolerance of the probiotic *L. rhamnosus* (Correa Deza et al., 2017). Polyphosphate gained further interest as a beneficial bacterial product, which participates in the maintenance of the gut barrier function by *L. casei* (Saiki et al., 2016) and by *L. brevis* (Segawa et al., 2011; Tanaka et al., 2015). This active molecule, derived from *L. brevis*, was further shown to supress intestinal inflammation and fibrosis in 2 models of induced colitis in mice (Kashima et al., 2015) and to inhibit colon cancer progression, in an animal xenograft model, through induction of cell apoptosis (Sakatani et al., 2016). In our work, the elevated concentrations of calcium-phosphate and of lactose in hyperconcentrated sweet whey are consistent with the observed accumulation of polyphosphate and of carbohydrates.

The proteomic analysis performed here is in agreement with the physiological data. Increase in sweet whey dry matter concentration led to accumulation of key stress proteins involved in bacterial adaptation. This includes proteins known to participate in the repair, protection, and turnover of macromolecules, proteins and nucleic acids. The proteomic signature of *L. casei* BL23 in hyperconcentrated sweet whey further includes proteins which participate in osmoadaptation. Indeed, over-expression of *oppA*, encoding an oligopeptide binding protein, confers enhanced resistance to salt, heat and bile stress in *L. salivarius* (Wang et al., 2015). Proteins involved in oxidative and metal stress remediation were also induced, in accordance with the observed multitolerance.

Proteins induced in hyperconcentrated sweet whey also included proteins involved in the envelop metabolism, such as yqhA and pepN. Accordingly, we observed modifications in *L. casei* BL23 morphology, including thinning of the cell wall and curvature of the bacteria in 30% sweet whey cultures. Thinning of the cell wall, as a result of hyperosmotic adaptation, was already described in *L. casei* BL23 (Piuri et al., 2005; Palomino et al., 2013), as well as in the soil bacterium *Bacillus subtilis*. High salt stress modifies its morphology, with a thinner and denser peptidoglycan cell wall in the presence of salt (Palomino et al., 2009). Osmoadaptation, as a result of osmotic stress sensing, indeed results in fluxes of water, which in turn cause modifications in water activity, intracellular volume, turgor pressure, cell wall deformation, and curvature (Morbach and Krämer, 2002).

In accordance with the observed accumulation of general and heat stress proteins such as groS and DnaK, hyperconcentrated sweet whey cultures of *L. casei* BL23 displayed enhanced tolerance to spray-drying. Interestingly, enhancement of the survival rate appeared dose-dependent, as 30% sweet whey afforded a higher survival than 20% sweet whey, and 20% a higher one than 5%. Indeed, the bottleneck of this process is the important heat stress imposed to the bacteria, and oxidative stress due exposure to air stream. Enhanced thermotolerance and accumulation of proteins known to participate in oxidative stress remediation, together with accumulation of protective intracellular compatible solutes, explain the toughness acquired by BL23 cultured in 30% sweet whey.

Production of dried lactic acid starters and of dried probiotics is a difficult task. It is risky, because of all the abiotic stresses that beneficial bacteria will undergo during drying and subsequent storage. It is compulsory because industrials will more and more ask for high quality dried products, whether as fermentation starters, or as probiotic ingredients. Indeed, the expected stability of live bacteria in the dried product should afford a loss of viability below 0.5 log during a 1-year storage at 25°C (personal communication from stakeholders of the probiotic industry). Culture conditions, pre-treatments, drying media and drying processes should favor the development of tolerance toward technological stresses related to drying and storage. They should also maximize tolerance toward digestive stresses, in probiotic applications. Both freeze-drying and spray-drying processing schemes should thus be improved in this aim. In the present work, we propose a new process, which improves *L. casei* BL23 stress survival through an osmo-induced multitolerance: this probiotic is thus prepared for both technological and digestive stresses. The use of a food-grade medium avoids harvesting and washing steps. The high osmolarity of dairy industry side-products triggers multitolerance. Its low water content reduces the need for energy. As a perspective, such a multitolerance induction by hyperconcentrated growth media could also improve survival during freeze-drying. As an alternative, hyperconcentrated food-grade media of vegetal origin, such as soy milk, can be used in a vegetarian option. Altogether, this work opens new perspectives for the industrial production of probiotics reputed as fragile bacteria, such as *L. casei* BL23.

## Author Contributions

SH, FG, CC, and JJ performed the experiments. YLL, RJ, and XC supervised the work. All the authors participated in the writing of the paper.

## Conflict of Interest Statement

The authors declare that the research was conducted in the absence of any commercial or financial relationships that could be construed as a potential conflict of interest.

## Footnotes

1 https://imagej.nih.gov/ij/

2 http://www.uniprot.org/taxonomy/543734

## Supplementary Material

FIGURE S1 The figure shows the abundance ration for the selected proteins. Proteins with a ration below 1.5 (0.18 in log 10 scale) were not taken into account.
