# Supplementary figures and images for: Growth in Hyper-Concentrated Sweet Whey Triggers Multi Stress Tolerance and Spray Drying Survival in Lactobacillus casei BL23: From the Molecular Basis to New Perspectives for Sustainable Probiotic Production
Source: Front Microbiol. 2018 Oct 22;9:2548. doi: 10.3389/fmicb.2018.02548 (PMC6204390; doi:10.3389/fmicb.2018.02548)

Abundance ratio: 30/5

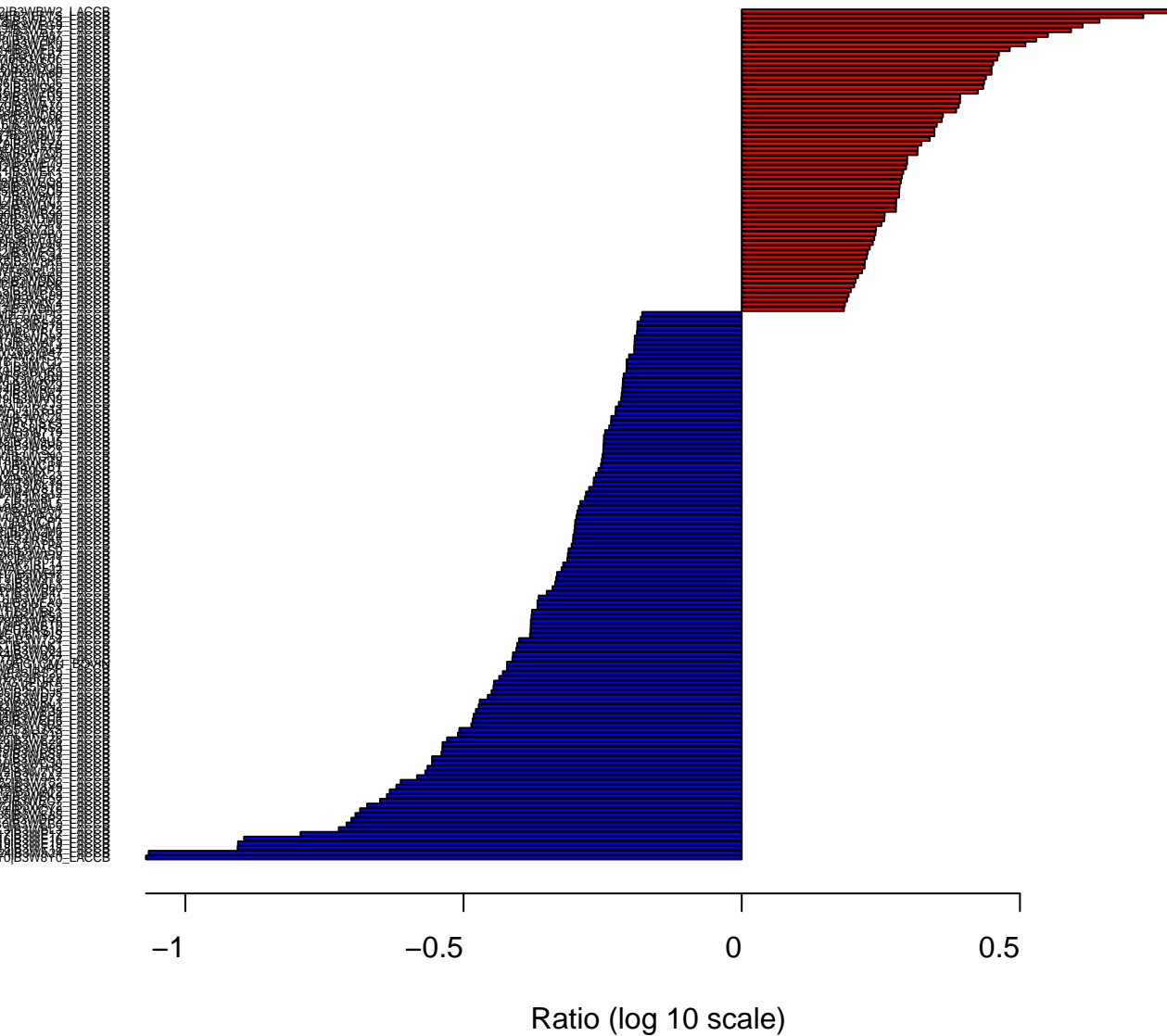

Supplement: FIGURE S1 — The figure shows the abundance ration for the selected proteins. Proteins with a ration below 1.5 (0.18 in log 10 scale) were not taken into account. [file Image_1.PDF]
